# Single graft “peninsula-style” transverse aortic arch replacement in patients with type A acute aortic dissection: case report

**DOI:** 10.1093/jscr/rjaf292

**Published:** 2025-05-09

**Authors:** Vladimir Belostotsky, Darko Bislimovski, Aleksandar Nikolic, Milan Milojevic

**Affiliations:** Department of Cardiovascular Surgery, Acibadem Sistina Hospital, Skupi 5A, 1000 Skopje, Republic of North Macedonia; Department of Cardiovascular Surgery, Acibadem Sistina Hospital, Skupi 5A, 1000 Skopje, Republic of North Macedonia; Department of Cardiovascular Surgery, Acibadem Sistina Hospital, Skupi 5A, 1000 Skopje, Republic of North Macedonia; Department of Cardiovascular Surgery, Acibadem Sistina Hospital, Skupi 5A, 1000 Skopje, Republic of North Macedonia; Department of Cardiothoracic Surgery, Erasmus University Medical Center, Dr. Molewaterplein 40, 3015GD Rotterdam, The Netherlands

**Keywords:** acute type A aortic dissection, DeBakey type II, aortic arch surgery, “peninsula-style” technique

## Abstract

Acute type A aortic dissection, despite treatment advances, remains a critical emergency with markedly high morbidity and mortality rates. The primary goals of immediate surgical intervention are to ensure survival, prevent severe complications, and avoid subsequent interventions. We present a case of a 55-year-old male who presented with new-onset chest pain, dyspnea, and hypotension. Emergent transthoracic echocardiography and subsequent computed tomography revealed an ascending aortic dissection. The patient underwent immediate surgical repair using a “peninsula-style” technique for transverse arch replacement with a single piece of Dacron graft, preserving continuity with the proximal descending aorta and performing routine aortic valve commissural resuspension. Following an uncomplicated postoperative course, he was discharged in a stable condition, and an 18-month follow-up CT showed no signs of aorta-related complications. This case report underscores the importance of having specialized thoracic aortic teams capable of using easily reproducible techniques, reducing operative time, and yielding reliable results.

## Introduction

Acute type A aortic dissection is a life-threatening surgical emergency requiring immediate intervention to prevent fatal complications. Successful outcomes depend on rapid diagnosis, efficient coordination within an experienced aortic team, and timely surgical management. While standard surgical approaches exist, complex cases often require tailored techniques to achieve optimal outcomes. This case highlights the successful application of a modified “Peninsula-Style” technique for DeBakey type II dissection, demonstrating how strategic operative planning and a multidisciplinary approach can enhance surgical precision, minimize circulatory arrest time, and improve long-term patient outcomes. Sharing such experiences contributes to the refinement of surgical strategies and the advancement of aortic arch repair techniques.

## Case report

**Figure 1:**
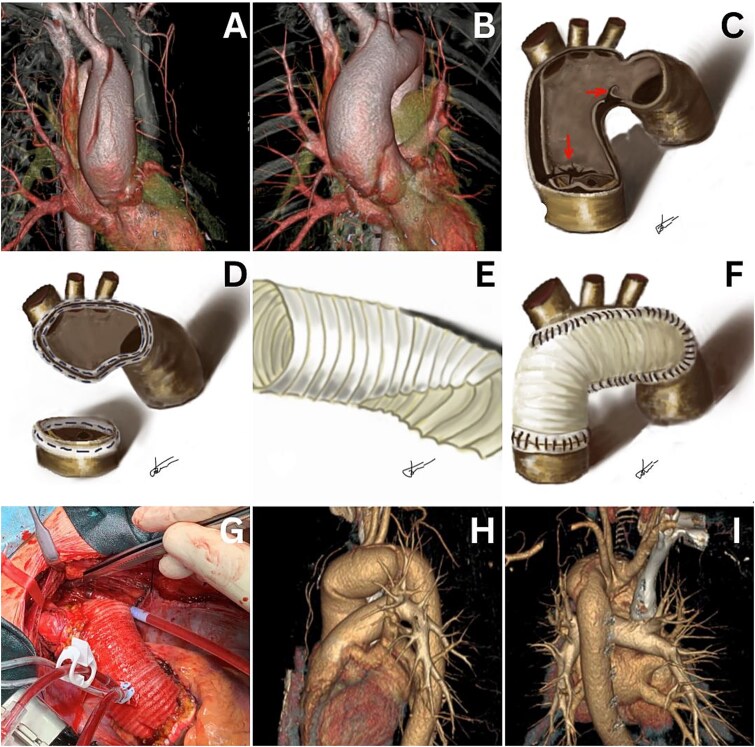
Computed tomography reveals a type A acute aortic dissection (A and B). Freehand drawings illustrate marked entry and reentry points (C), aortotomy sites for anastomoses (D), and tailoring of the Dacron graft (E and F). The final intraoperative treatment result is shown (G). Computed tomography images demonstrate good treatment results at the 18-month follow-up in the outpatient clinic (H and I).

A 55-year-old male with uncontrolled hypertension presented to the emergency department of Acibadem-Sistina Hospital in Skopje, North Macedonia, with new-onset chest pain, dyspnea, and hypotension. Given the patient’s hemodynamic instability, a bedside transthoracic echocardiography was promptly performed, revealing acute type A aortic dissection (E1M0) and moderate aortic valve insufficiency. Subsequently, a computed tomographic angiogram (Fig. 1A and B) confirmed Acute Stanford type A, DeBakey type II dissection and provided comprehensive anatomical detail essential for surgical planning, and the patient was transferred to the operating room.

Cardiopulmonary bypass (CPB) was initiated using an arterial line through the right axillary artery [8 mm knitted double-velour Dacron graft (Maquet, Wayne, NJ)] for both CPB and selective antegrade cerebral perfusion, with venous return through the right atrial appendage. The procedure was performed in moderate systemic hypothermia (27°C) using the open distal anastomosis technique. Aortotomy revealed visible entry and re-entry tears (Fig. 1C).

A single long suture line was placed from near the ligamentum arteriosum in the distal arch to the innominate artery take-off on the along the greater curvature of the aortic arch (Fig. 1D). A custom-tailored 28 mm woven double-velour Dacron graft was used to match the peninsula shape on the greater curve of the arch (Fig. 1E and F). The proximal anastomosis was performed using routine techniques during rewarming, including aortic valve commissural resuspension. Both anastomoses were reinforced with a Teflon-felt “sandwich” technique and BioGlue® applied with suction assistance [1].

The duration of circulatory arrest with antegrade cerebral perfusion was 52 min, aortic cross-clamp time was 95 min, and CPB time was 132 min. An intraoperative photograph shows the completed “peninsula-style” transverse arch repair (Fig. 1G). The postoperative period was uneventful, and the patient was discharged on the sixth postoperative day. Eighteen months postoperatively, the patient remains in good condition without any complaints (Fig. 1H and I).

## Discussion

The anatomical confinement of the dissection to the aortic arch (DeBakey type II) with the distal fenestration just below the left subclavian artery, prompted the selection of “Peninsula-Style” technique described by Akinobu Itoh *et al.* [2] as the most appropriate surgical approach for this patient. Although this technique was initially used for patients with dilatation of the aortic arch and intact brachiocephalic vessels, we adapted it for our patient whose brachiocephalic vessels were partially dissected and restored using Teflon-felts.

This operation is more straightforward compared to the island (or en bloc) technique or the Kazui technique [3], and it may help avoid complications associated with increased circulatory arrest times and potential bleeding from the Carrel patch, as noted by Peter Chiu and co-authors [4]. We used only one tubular vascular graft, unlike other techniques that use two vascular grafts with subsequent anastomosis [5]. However, we acknowledge that the circulatory arrest time of 52 min is on the higher end, even with selective antegrade cerebral perfusion. A conventional zone II arch replacement, particularly with prefabricated branched grafts, can often result in shorter arrest times and facilitate more extensive resection of diseased tissue. However, in this case, the dissection was distally limited, and the peninsula-style technique offered a favorable balance between adequate resection and procedural efficiency. While zone II replacement remains the standard in cases of more extensive disease, the peninsula-style approach may be preferable in selected patients, particularly when the dissection is localized, supra-aortic vessels are repairable, and there is a need to minimize operative time or technical complexity.

In conclusion, the peninsula-style approach allowed the use of one vascular prosthesis and only two anastomoses, reducing operative time withouth compromising efficacy. The patient experienced no postoperative complications and achieved excellent early and 18-month outcomes, supporting the value of this technique in appropriately selected cases.
